# Special Hospitals and Their Advertisements

**Published:** 1912-06-08

**Authors:** 


### The Hospital

The Workers' Newspaper of Administrative Medicine and Institutional Life, Administration, National Insurance and Health.

No. 1353, Vol. LII. SATURDAY, JUNE 8, 1912. PRICE ONE PENNY.

### SPECIAL HOSPITALS AND THEIR ADVERTISEMENTS.

At the present moment an attempt is being made to arouse resentment in the ranks of the medical profession against the General Medical Council on account of certain proceedings taken by that body in dealing with a complaint preferred by the British Medical Association against Mr. J. Forrest, M.B. The statement of Mr. Forrest's case, lately circulated in pamphlet form, is very largely an attack upon some voluntary hospitals as at present conducted. Assuming, for the sake of argument, that all Mr. Forrest's statements are perfectly accurate, and that no material portion of the case against him has been suppressed in his account of it, a perusal of his defence leaves the impression that a less provocative manner might have removed many of the difficulties which he got into with the General Medical Council; and even that a little more tact in his campaign might have prevented the proceedings altogether.

The facts, as set out by Mr. Forrest, are briefly these. He is an Edinburgh graduate who, after five years in general practice, decided to come to London for more hospital experience. For five years he filled various resident and non-resident offices at special hospitals, devoting himself mainly to eye, ear, nose, and throat work. This period of study convinced him that a conspiracy exists to debar the general practitioner from proper opportunities of becoming proficient in these sciences, and to keep them as a close preserve for specialists. He says that the managements of the special ear, throat, and eye hospitals deliberately set out to attract middle-class patients, who may be unable to pay a specialist's high fees but can yet contribute substantially to the funds of the hospitals. He indicts these hospitals as a class, and gives a particular instance which came under his notice at the Throat Hospital, Golden Square. If this specific charge is true, the episode can only be described as most discreditable; but until the hospital has entered a defence, judgment must necessarily be in abeyance. As for the general, as opposed to the particular, accusation, our experience leads us to believe that there is unfortunately something in it, much as it is to be deplored. Mr. Forrest supports his contentions by pointing out that some of the ear and throat hospitals advertise extensively in *Lloyd's News* and similar papers, soliciting the patronage of paying patients; and he seems here to have laid his finger on a form of hospital abuse which undoubtedly exists. His remarks on the inaction of the ethical committee of the British Medical Association in this matter, if caustic, are still not devoid of some foundation. It is impossible to give all Mr. Forrest's allegations in detail; but it is to be hoped that some rebuttal, or else some indication of official repentance, may be forthcoming.

Filled with discontent at the system under which he had been working, Mr. Forrest instituted a private clinique for patients suffering from diseases of the special sense organs already mentioned. At this institution patients were required to pay small fees; half a guinea for the initial consultation, and five shillings thereafter for each one subsequently. It is also stated that no financial profit was sought after, and that the remuneration of the two medical officers was not to exceed £100 per annum. Operative work was undertaken at rates proportional to the consultation fees already mentioned. Advertisements were circulated soliciting patients; the names of the surgeons were, it is said, not given in these advertisements. Prescriptions were written, and patients were encouraged to remain under the care of private practitioners, and to attend occasionally for supervision of such treatment. These activities were promptly denounced to the General Medical Council by the British Medical Association, and Mr. Forrest was called to account before the former body. Filled with a *sæva indignatio* at what he conceived to be unfair treatment, he attacked in his defence both his accusers and the hospital system which had excited his disapproval. After part of the hearing, he asked for the removal of his name from the Register. In the comments which he now publishes, the defendant impeaches the judicial fairness of the tribunal before which he appeared; and denounces, left and right, specialists, hospitals, Council, and Association. This tirade is delivered with no mean ability, though in somewhat intemperate language; doubtless it is not easy to remain calm while smarting under a sense of injuries such as those which Mr. Forrest evidently feels.

We have recounted the contentions of his pamphlet at some length because it is clear that one of the issues at stake was never thrashed out during the hearing of the case. The defendant was accused of advertising his clinique; and his answer was, in substance, "Others are just as bad." This, of course, is no excuse for wrongdoing, if such there was; but, from an institutional and public point of view, the charge should be investigated, and this was, as it happens, somewhat half-heartedly admitted by the (then) Medical Secretary of the British Medical Association. Until the promised inquiry is undertaken, judgment must be in suspense. His statements are so definite that for their own credit's sake the special hospitals must reply to them; and if they fail to clear themselves, both medical and lay opinion is likely to side against them. The British Medical Association is equally involved, and should take the matter up promptly and impartially.

